# Superior vena cava obstruction post laparoscopic cholecystectomy: A case report

**DOI:** 10.1002/ccr3.1930

**Published:** 2018-11-20

**Authors:** Simbarashe Gift Mungazi, Rudo Gwini, Munyaradzi S Magara

**Affiliations:** ^1^ Faculty of Medicine, Department of Surgery and Anaesthetics National University of Science and Technology Bulawayo Zimbabwe; ^2^ Faculty of Medicine, Department of Medicine National University of Science and Technology Bulawayo Zimbabwe

**Keywords:** anticoagulation, laparoscopic cholecystectomy, superior vena cava obstruction, thrombosis

## Abstract

Clinicians should have a high index of suspicion of superior vena cava obstruction in a patient presenting with painful lateral neck and ipsilateral chest swelling post laparoscopic procedures. High doses of Clexane can be used as a substitute for thrombolytic therapy where it is contraindicated.

## 1 INTRODUCTION

Superior vena cava obstruction is a manifestation of benign or malignant disease obstructing return of blood flow through the superior vena cava (SVC). We present the case of a 33‐year‐old, obese female patient with an unusual complication of superior vena cava obstruction 3 days post laparoscopic cholecystectomy.

Superior vena cava obstruction is a manifestation of benign or malignant disease obstructing return of blood flow through the superior vena cava (SVC).^1^ Majority of cases of acute onset superior vena cava (SVC) obstruction is due to thrombosis caused by central venous catheters. Most other causes of acute obstruction are benign.^2^ Paget‐Schroetter is a symptomatic compression of the subclavian vein as it passes through the narrow space between skeletal and muscular component of the shoulder girdle.^3^ Malignancies generally cause a gradual obstruction, and the commonest of these is bronchogenic carcinoma.^2^ Deep vein thrombosis is a well‐known complication post conventional open surgery. However, it is not a common complication of laparoscopic procedures although a few cases of deep vein thrombosis post laparoscopic procedures have been documented.^4^

## 2 CASE REPORT

A 33‐year‐old, obese female patient presented with a 3‐day history of swelling of the right side of the neck, right chest and upper limb pain and shortness of breath when talking. This was 3 days after a laparoscopic cholecystectomy for calculous cholecystitis. She also had a lower segment cesarean section 6 weeks prior to the cholecystectomy. She did not have a history of heavy manual activity involving the upper limbs. There was no history of trauma. She had no history of smoking, chronic illnesses, or autoimmune conditions. There was no positive personal or family history of malignancy. The patient had received Clexane 40 mg subcutaneously once daily, post operatively to prevent deep vein thrombosis after laparoscopic cholecystectomy.

**Figure 1:**
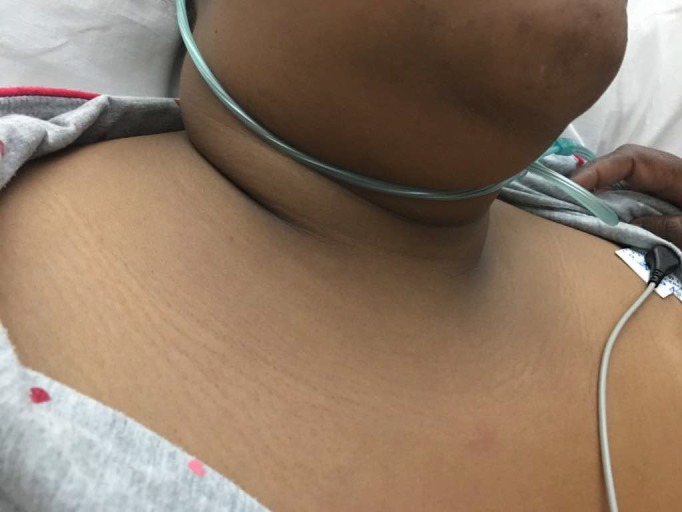
Right neck swelling

**Figure 2:**
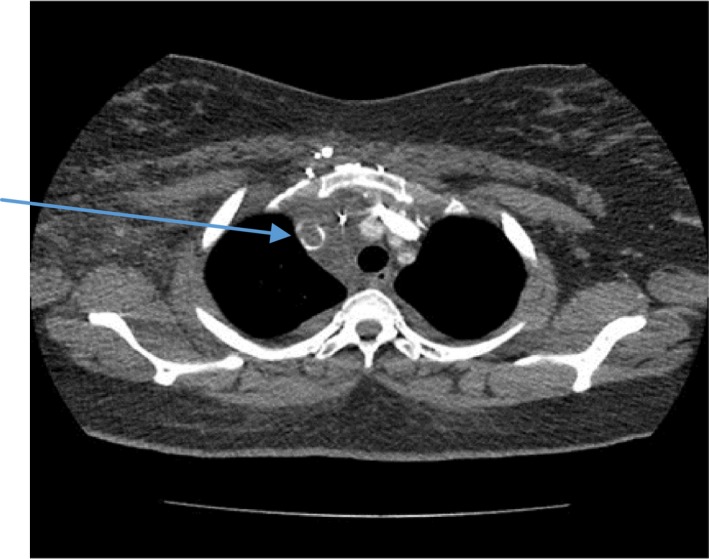
Thrombi in the brachiocephalic vein (arrow)

**Figure 3:**
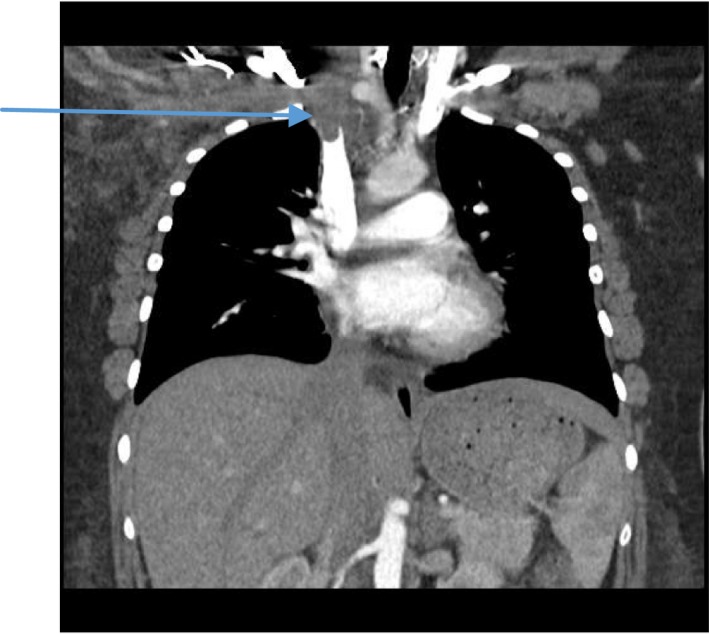
Thrombi extending into the superior vena cava (arrow)

**Figure 4:**
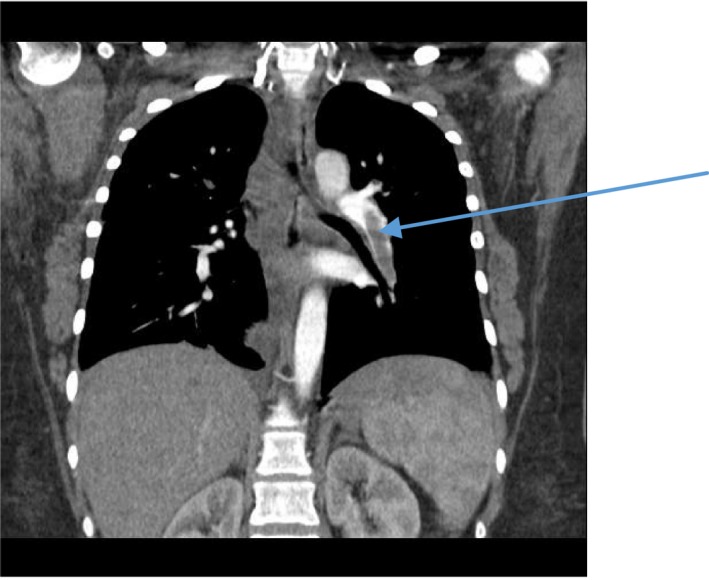
Pulmonary embolism (arrow)

On examination, the patient was in pain with a swelling on the right side of the neck and right upper limb (Figure 1). The vitals were Temp 36.2°C, blood pressure 157/101 mm Hg, heart rate 72/minute, respiratory rate 36 breadths per minute, and saturation of 100% on free air. Her weight was 95 kg with a body mass index (BMI) of 40 kg/m^2^. She had tenderness of the right side of the chest, right upper limb, and right side of the neck. The chest had normal vesicular breath sounds. There were no breast lumps. There was no limb swelling. The abdomen was normal, and the rest of the examination was unremarkable. Investigations done showed the following; full blood count: hemoglobin 9.5 g/dL white cell count 12.5 cells/mm^3^, platelet 390 × 10^3^/μL, mean corpuscular volume 77. The urea and electrolytes, clotting profile, serum cholesterol and triglycerides, and glycosylated hemoglobin were normal. Antiphospholipid antibodies were negative. Protein C and protein S were not done as they could not be done at the local laboratory. The computed tomography (CT) scan of the chest showed (a) extensive soft tissue swelling of the right neck extending to the mediastinum, (b) thrombosis of the brachiocephalic vein extending into the Superior vena cava (Figures 2 and 3), and (c) bilateral pulmonary artery branches filling defects consistent with embolism (Figure 4). An ultrasound of the abdomen and Doppler scan of both lower limbs were normal.

The patient was admitted in high dependent unit (HDU), put on oxygen per nasal prongs at 4 L/min, Clexane (Emcure^®^, Pune, India) 80 mg 12 hourly, Amlodipine 5 mg once daily, analgesia, strict bed rest, and propped up. Because of the recent surgery, there was concern on the use of antithrombolytics. The swelling subsided, and the pain decreased. The patient was started on physiotherapy. The patient did well and was discharged after 2 weeks on Clexane 40 mg subcutaneously daily and analgesia. On review 2 weeks’ post discharge, the patient had fully recovered. A repeat CT scan showed no presence of any thrombi in the right brachiocephalic vein as well as the absence of pulmonary embolism. Unfortunately, we could not monitor the antifactor Xa assay for anticoagulation therapy. This test is not routinely done at our center.

## 3 DISCUSSION

Superior vena cava (SVC) obstruction is a serious condition which can be caused by a benign or malignant disease. In one case series, malignancy was by far the most common etiology.^2^, ^5^ Laparoscopic procedures have been shown to reduce the number of complications and reduce hospital stay compared to open surgery. However, a few cases of DVT have been documented.^4^ To our knowledge, SVC obstruction post laparoscopic procedure has not been documented.

A thorough history, physical examination and investigations on our patient were done to identify risk factors for developing SVC obstruction. Apart from obesity, no other cause could be linked to the development of SVC obstruction. Furthermore, our patient did not have a first rib anomaly as described by Paget‐Schroetter.^6^ In addition, the subclavian vein of our patient was not involved with thrombi. A central venous catheter was not used on the patient on both previous operations. A high BMI in our patient is a known risk factor for deep vein thrombosis.^7^ There is a correlation of BMI and DVT in patients with a genetic predisposition, indicating that, this should be taken into consideration when conducting laparoscopic surgery. In a prospective randomized study done to evaluate the occurrence of deep vein thrombosis (DVT) in laparoscopic cholecystectomy patients, results showed no difference in the group that had received nadroparin and placebo.^4^ Despite giving our patient prophylactic doses of Clexane (LMWH), the patient went on to develop an unusual of SVC obstruction. This case is being reported to raise awareness of thromboembolic phenomenon despite giving LMWH post laparoscopic cholecystectomy.

Anticoagualtion, thrombolytics, and thrombectomy or atherectomy catheters have also been used during or following stent implantation although their use remains primarily empiric. Percutaneous treatment of SVC obstruction offers patients hope for prompt and dramatic relief from the symptoms of SVCS.^1^ SVC stents have been used in patients in whom the condition failed to respond to traditional therapy or in whom symptoms recurred after such therapy. Relief of symptoms has been demonstrated in more than 90% of patients with stents.^2^ Our case, being in a resource‐limited environment, treatment options were limited. This case demonstrates that SVC obstruction can be managed using high‐dose LMWH where the use of thrombolytics or surgery for SVC obstruction is contraindicated or unavailable. Our patient was 3 days post‐surgery, and the role of other treatment modalities such as thrombolytics was unsafe. The cause for the SVC obstruction on our patient could be linked to her high BMI though genetic testing could not be done to establish this. Therefore, the cause of SVC still remains unknown. Literature does note such occurrences.^8^

## 4 CONCLUSION

Despite laparoscopic procedures being associated with minimal complications such as DVT and prolonged hospital stay, it is prudent to observe all patients for development of DVT, pulmonary embolism, and SVC obstruction. Clinicians should have a high index of suspicion of superior vena cava obstruction in a patient presenting with painful lateral neck and ipsilateral chest swelling. Even when recent surgery is a contraindication to the use of thrombolytics, the use of high doses of Clexane brings dramatic relief. An early diagnoses and appropriate treatment will result in a good outcome.

## CONSENT

Written informed consent was obtained from the patient for publication of this case report and accompanying images. A copy of the written consent is available for review.

## CONFLICT OF INTEREST

No conflict of interest.

## AUTHOR CONTRIBUTION

SGM: designed the case report, involved in subject research, obtained the consent, and wrote the manuscript. RG: designed the case report, involved in subject research, and edited and wrote the manuscript. MSM: designed the case report, edited and wrote the manuscript.

## ETHICAL APPROVAL

Ethical approval was exempted by our institution. A written consent from the patient was obtained.

## References

[1] Hochrein J , Bashore TM , O’Laughlin MP , Harrison J . Percutaneous stenting of superior vena cava syndrome: a case report and review of the literature. Am J Med. 1998;104(1):78‐84. 9528723 10.1016/s0002-9343(97)00345-8

[2] Gunaratne A . Acute superior vena cava obstruction causing total airway obstruction in the anaesthetic recovery room. Anaesth Pain Intensive Care. 2009;13(1):25‐27.

[3] Sanghavi ST , Vardhachary KS , Hoda A . Paget‐Schroetter syndrome. J Postgrad Med. 1983;29:175‐176. 6655607

[4] Schaepkens Van Riempst JT , Van Hee RH , Weyler JJ . Deep venous thrombosis after laparoscopic cholecystectomy and prevention with nadroparin. Surg Endosc. 2002;16:184‐187. 11961636 10.1007/s004640090048

[5] Chen JC , Bongard F , Klein SR . A contemporary perspective on superior vena cava syndrome. Am J Surg. 1990;160(2):207‐211. 2382775 10.1016/s0002-9610(05)80308-3

[6] Alla VM , Natarajan N , Kaushik M , Warrier R , Nair CK . Paget‐schroetter syndrome: review of pathogenesis and treatment of effort thrombosis. West J Emerg Med. 2010;11(4):358‐362. 21079709 PMC2967689

[7] Klovaite J , Benn M , Nordestgaard BG . Obesity as a causal risk factor for deep venous thrombosis: a Mendelian randomization study. J Intern Med. 2015;277:573‐584. 25161014 10.1111/joim.12299

[8] Tilney NL , Griffiths H , Edwards EA . Natural history of major venous thrombosis of the upper extremity. Arch Surg. 1970;101(6):792‐796. 5489306 10.1001/archsurg.1970.01340300148026

